# Neuromodulation for Adjunctive Treatment in Postmastectomy Pain Syndrome

**DOI:** 10.7759/cureus.47827

**Published:** 2023-10-27

**Authors:** Kennedy Kirkpatrick, Jay D Shah, Krishna Shah

**Affiliations:** 1 Anesthesiology, Baylor College of Medicine, Houston, USA; 2 Anesthesiology and Interventional Pain, Baylor College of Medicine, Houston, USA

**Keywords:** postmastectomy pain syndrome, dorsal root ganglion, neuromodulation, spinal cord stimulator, post mastectomy pain syndrome, pmps

## Abstract

Postmastectomy pain syndrome (PMPS) affects nearly half of patients who undergo mastectomy to treat breast cancer. As the survival rate of breast cancer increases with advancements in treatment, the incidence of PMPS is also increasing. Patients with PMPS can experience unrelenting, chronic pain refractory to traditional management with oral pharmacotherapy in conjunction with nonpharmacologic treatment (physical therapy, transcutaneous electrical nerve stimulation (TENS)). Neuromodulation is an emerging treatment modality for numerous chronic pain conditions. This case report highlights the tremendous success of spinal cord stimulator placement for a patient with PMPS.

## Introduction

Breast cancer is the most common cancer diagnosed globally, according to the American Cancer Society [1]. A cornerstone of breast cancer treatment involves surgical excision with either mastectomy or lumpectomy with radiation. Postoperative pain at least three months following mastectomy occurs in 36%-47% of patients and is referred to as postmastectomy pain syndrome (PMPS) if it is neuropathic in nature and involves the anterior surface of the chest axilla, shoulder, or upper half of the arm [2,3]. Postmastectomy pain syndrome is thought to evolve from entrapment or damage to the intercostobrachial nerve during axillary node dissection or surgical manipulation, resulting in sensitization of peripheral nociceptors and ectopic neural activity [3]. This condition causes significant morbidity among mastectomy patients, and no gold-standard treatment exists. Generally, physical therapy and weight-bearing exercises are trialed first, followed by pharmacologic management with ibuprofen or non-steroidal anti-inflammatory drugs (NSAIDs), tricyclic antidepressants (TCAs), serotonin-norepinephrine reuptake inhibitors (SNRIs), and gabapentinoids. Topical medications (capsaicin, topical morphine), infusions of ketamine, and opioids have also been documented in treatment algorithms. When noninvasive management and pharmacologic agents fail to provide pain relief, interventions such as transcutaneous electrical nerve stimulation (TENS), peripheral nerve blocks, radiofrequency ablation, and neuromodulation are logical next steps with growing supporting literature in neuropathic pain conditions.

Neuromodulation is an emerging approach over the last decade being used for more definitive treatment of chronic pain syndromes. This procedure involves targeted electrical stimulation of peripheral and central nervous system neuronal firing patterns to increase levels of pain-modulating neurotransmitters and suppress hyperexcitable pain-driving neurotransmitters. Specifically, the generation of electric fields between electrodes in the epidural space changes the electrical potentials of surrounding tissues, triggering action potentials in excitable areas and activating large dorsal column axons. Electrical charge is delivered using various waveforms differentiated between pulse amplitude, width, and frequency, which, in combination, deliver a charge to the targeted nerve area. It is not currently understood which fibers are activated to achieve maximal pain relief or how activation patterns change with chronic spinal cord stimulation (SCS). Currently, it is thought that SCS promotes the activation of gamma-aminobutyric acid (GABA) and adenosine-1 receptors, leading to pain modulation [4].

Neuromodulation has been used scarcely, but successfully, for PMPS pain. The two types of neuromodulation that have shown efficacy in chronic pain syndromes include traditional SCS (t-SCS) and dorsal root ganglion (DRG) stimulation. Here we describe a successful trial and implantation of a dorsal column spinal cord stimulator in a patient experiencing PMPS pain that was previously refractory to oral medications, physical therapy, and targeted nerve injections.

## Case presentation

A 57-year-old female presented to the interventional pain clinic with right-sided neuropathic breast pain that started at the end of October 2019 following treatment for breast cancer. The patient was initially treated with lumpectomy for right breast ductal carcinoma in situ (DCIS); however, after one year, the patient had a 2.5cm growth at her lumpectomy site showing a human epidermal growth factor receptor 2-positive (HER2+) invasive ductal carcinoma, treated with mastectomy. The patient received a right breast reconstruction with a deep inferior epigastric perforator flap, followed by 20 sessions of breast radiation, causing severe breast pain. The pain was described as diffuse, burning pain along her right breast, axilla, and right upper extremity, with concurrent allodynia in these areas as well. She had previously tried physical therapy and desensitization without relief. Furthermore, a thoracic medial branch block was attempted once with minimal, short-term relief. Her pain was managed with Lyrica 75 mg twice daily, but her symptoms continued to limit her daily activities. She noted that the nerve pain became much more severe if she missed even one dose of Lyrica.

As the pain continued to cause increased functional limitations and neuropathic medications had not been successful for more than 12 months, the decision was made to trial SCS implantation. Before trial implantation, a cervical MRI was obtained and showed no acute findings with age-appropriate changes. Her baseline pain levels before SCS implantation ranged from 6/10 to 10/10, with an Oswestry Disability Index (ODI) of 42%, which is considered a moderate disability.

**Figure 1:**
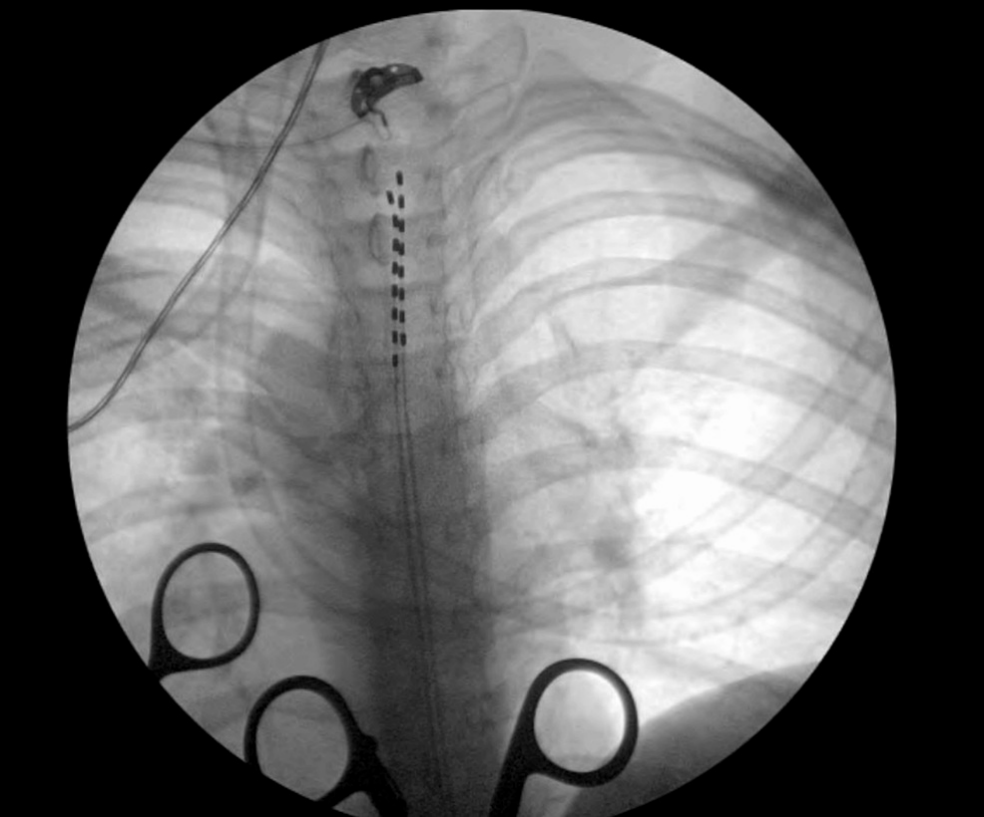
Radiographic evidence of SCS implantation with lead placement SCS: spinal cord stimulator

For trial implantation, epidural access was obtained at the T12/L1 level, and neurostimulator electrode leads were placed at the top of thoracic T2 and middle of T3 vertebral levels, as seen below in Figure 1.

Differential target multiplexed (DTM) SCS was used. Following a seven-day trial implantation, she experienced over 80% pain relief during the trial implantation as well as significant improvements in mood and functional ability. Notably, the patient did not require any neuropathic pain medication during the trial period. A permanent SCS was implanted at the same vertebral levels as the trial stimulator. Pain scores were reassessed after permanent implantation at one week, three months, and six months, with the patient continuing to experience significant pain relief and significant improvement with an ODI score of 0% at the six-month follow-up, which is consistent with no disability. There was a drastic improvement in pain as well as quality of life at the six-month follow-up for our patient. In addition, complete cessation of both NSAIDs and neuropathic pain medications was achieved at six-month follow-up (Table 1).

**Table 1:** Pain and quality of life pre-SCS implantation and six months post-SCS implantation NSAIDs: non-steroidal anti-inflammatory drugs; SCS: spinal cord stimulator This table compares quality of life scores, pain scores, and medication usage prior to permanent SCS implantation and after SCS implantation. Significant improvements in both the ODI index score and quality of life were seen. Cessation of both NSAIDs and neuropathic medications was achieved at the six-month follow-up.

	Before implantation	Six months after implantation
Oswestry Disability Index (ODI) Score	42%	0%
Pain	6	0
Neuropathic pain medication dose	75mg Lyrica	None
NSAID dose	PRN, daily	None
Sleep score	Pain with medications, <4 hours of sleep	No effect on sleep
Social life	Restricted social life due to pain	No effect on social life
Travel	Trips limited to <1 hour	No effect on travel
Lifting	Able to lift light weights	Able to lift heavy weights without pain
Homemaking	Pain prevents anything but light activity	No limitation on activity
Walking	No limitations	No limitations
Sitting	No limitations	No limitations
Standing	No limitations	No limitations

## Discussion

Postmastectomy pain syndrome can be challenging to treat as current treatment options are limited and many have sparse supporting literature. Often, patients need to heavily rely on neuropathic oral medications such as gabapentin and pregabalin. However, these medications are not always successful and also come with unwanted side effects such as drowsiness, altered mental status, and weight gain. Alternatively, peripheral nerve blocks have utility but are difficult to obtain long-lasting pain relief.

Neuromodulation has been FDA-approved for various neuropathic pain conditions, including post-laminectomy pain syndrome, complex regional pain syndrome, and diabetic neuropathy. Absolute contraindications to SCS placement include previous dorsal root entry zone surgery or disruption, critical central canal stenosis, neurologic deficit with surgically correctable pathology, spine instability or deformity at risk for progression, need for future MRI or implantable cardioverter defibrillator (ICD) placement, pregnancy, severe cognitive impairment interfering with device operation, unacceptable living situation or social environment, and active substance use disorder. Relative contraindications that would preclude SCS placement include anatomic conditions (epidural scarring from prior surgery, severe spondylolisthesis with stenosis, scoliosis creating difficulty with lead steering), medical comorbidities (untreated infection, anticoagulant or antiplatelet therapy), and psychosocial factors including reduced functional capacity, somatoform pain disorder, and significant psychosocial characteristics [2]. We recommend adequate history-taking to rule out any factors that could hinder SCS efficacy. In addition, shared decision-making is imperative, as permanent implantation incurs significant responsibility from a patient perspective.

Given the large neuropathic component of PMPS, we feel trialing a spinal cord stimulator among patients who have failed conventional therapy to assess the improvement in a patient’s ODI is warranted. Spinal cord stimulation involves the development and modulation of electric fields between electrodes, leading to an increased level of pain, attenuating neurotransmitters, and suppressing hyperexcitable neurons and neuronal pathways. Traditional SCS targets large-diameter dorsal column neurons, which in turn can inhibit pain signaling. Dorsal root ganglion stimulation, another form of spinal cord stimulation, directly targets the primary cell bodies of the dorsal root ganglion and has also shown efficacy in the management of chronic neuropathic pain. Dorsal root ganglion stimulation has increased root specificity, allowing for more targeted pain attenuation. Trials of neuromodulation for chronic neuropathic pain continue to grow; however, further data are needed to determine efficacy among PMPS patients.

A seven-patient case series done at a major US cancer center for PMPS patients found that 85% of patients achieved >50% pain relief with neuromodulation [5]. A case report of a PMPS patient from Germany who underwent DRG stimulation was followed over four years, with her medication requirements decreasing by 50% and a four-point numerical rating scale (NRS-11) pain scale score decreasing [6]. These cases highlight the importance of patient selection in the utility of neuromodulation for PMPS. The patients across these cases had all trialed oral medications, physical therapy, and other minimally invasive procedures. Also, these patients all had well-localized pain syndromes that could be targeted by traditional SCS or DRG stimulation.

As this case series showed, there is significant inter-patient variability as it relates to the technical aspects of spinal cord stimulator implantation. Lead placement in these cases varied from as high as C3 to as low as T8. The authors of this case series also used a variety of stimulators, including burst SCS and tonic SCS. Further investigation would need to be conducted to elucidate how lead placement or SCS type may impact outcomes in PMPS. However, the manifestation and localization of symptoms often vary between patients in PMPS, so the provider's ability to tailor therapy specific to the nature, location, and severity of pain with SCS is essential. In our case, we opted to place leads at the T2/T3 levels and use DTM stimulation. A 2021 multicenter trial of chronic low back pain patients comparing DTM stimulation to traditional SCS showed similar safety profiles and a significantly higher responder rate (statistical noninferiority and superiority) within the DTM group as compared to the SCS group [6]. Differential target multiplexed stimulation, first established in animal models in 2016, is theorized to have more efficacy in more complex pain syndromes due to the differential signals modulating glial cell gene expression more effectively than a single signal (seen from t-SCS programs) [7]. These studies underscore the importance of evaluating the range of neuromodulation techniques for our patient population, as outcomes may vary between chronic low back pain patients and those with PMPS.

Given the unique pain patterns that follow a mastectomy and radiation treatment, we would stress the importance of mapping the stimulator during the trial, as lead placement will likely vary from patient to patient. As the pain in PMPS is unilateral, we also recommend lead placement midline or slightly off midline to the ipsilateral side of the symptom occurrence. We recommend close follow-up throughout the trial period to determine the appropriate programming of the SCS. Aside from pain relief, we looked for other factors, including sleep, mood, a decrease in oral medications, and overall well-being, in determining a successful trial and discussion of permanent implantation.

**Figure 2:**
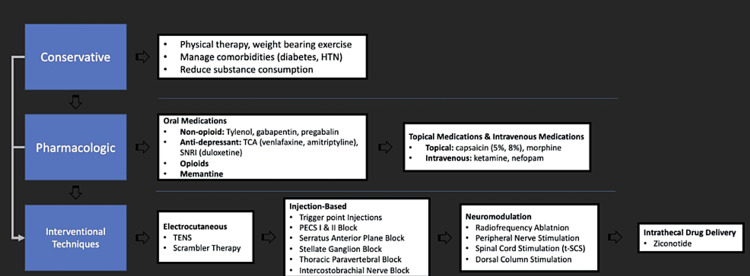
Suggested PMPS treatment algorithm This figure highlights the suggested algorithm recommended for PMPS pain following a review of current literature. PMPS: postmastectomy pain syndrome; HTN: hypertension; TCA: tricyclic antidepressant; SNRI: serotonin-norepinephrine reuptake inhibitors; TENS: transcutaneous electrical nerve stimulation; PECS: pectoral nerve

Limitations of this case report include the lack of robust clinical trials for the use of neuromodulation in PMPS patients. In addition, given there is no control group to compare to, less invasive options should be trialed first, as described below in Figure 2, as treatment response varies significantly from patient to patient.

This treatment modality should be considered in patients who are experiencing continued neuropathic pain after failing conservative therapies including desensitization, physical therapy, oral neuropathic medications, and peripheral nerve blocks. A recent review on the use of neuromodulation in chronic post-cancer pain (i.e., PMPS, chest wall pain, etc.) underscored the importance of further integration of neuromodulation in these debilitating and often difficult-to-treat conditions. To date, approximately 10 successful implantations of SCS for PMPS treatment have been reported in the literature [8].

Our patient’s successful implantation eliminated oral medications completely and, more importantly, significantly improved her quality of life. She has returned to running and traveling, and she finally feels that she has healed from her breast cancer battle. We anticipate annual follow-up for this patient unless more frequent follow-up is needed or complications arise.

## Conclusions

To the best of our knowledge, this case represents an early successful use of SCS in the management of PMPS. Spinal cord stimulation has incredible potential in the management of neuropathic pain among PMPS patients, targeting specific vertebral levels with proper lead placement. Advances in SCS technology, including the use of dorsal column SCS and burst SCS, highlight the promise of further research and investigation into the use of neuromodulation to treat chronic postmastectomy pain.
